# Supplementary material for: Effects of aqueous and ethanolic extracts of Chinese propolis on dental pulp stem cell viability, migration and cytokine expression
Source: PeerJ. 2024 Dec 18;12:e18742. doi: 10.7717/peerj.18742 (PMC11662894; doi:10.7717/peerj.18742)
Supplement: Supplemental Information 2 [file peerj-12-18742-s002.docx]

**Effects of Aqueous and Ethanolic Extracts of Chinese Propolis on Dental Pulp Stem Cell Viability, Migration and Cytokine Expression**

Ha Bin Park, Yen Dinh, Pilar Yesares, Jennifer L. Gibbs, Benoit Michot

**Supplementary figure S1: Amplification efficiency of Taqman primers.**

The efficiency of the primers was determined using the CT slope method with 6 concentrations of cDNA from 0.7 to 25 ng/uL (2x dilution factor). The efficiency of each primer is indicated on the graphs.

**Supplementary figure S2: Effects of AEP and EEP solvent on DPSC viability.** DPSCs were treated for 7 days with control media (Ctrl), dilutions of AEP solvent (A) or EEP solvent (B), and cell viability was evaluated using MTT assay. The dilutions of solvent correspond to the quantity of solvent in each dose of the respective propolis extracts (1/3 dilution, corresponds to the solvent dilution contained into the propolis extract solution at 33 mg/mL). Dash line indicates 30% decrease in cell viability, which represent the level of cytotoxicity. ** p<0.01, compared to control group, Dunnett’s test, 6 replicates from n=2 independent cell cultures.
